# The role of the platelet pool of Plasminogen Activator Inhibitor-1 in well-controlled type 2 diabetes patients

**DOI:** 10.1371/journal.pone.0267833

**Published:** 2022-08-31

**Authors:** Karin Mossberg, Josefin Olausson, Emanuel Fryk, Sverker Jern, Per-Anders Jansson, Helén Brogren

**Affiliations:** 1 Institute of Medicine, Sahlgrenska Academy, University of Gothenburg, Sahlgrenska University Hospital, Göteborg, Sweden; 2 Department of Public Health and Community Medicine, Sahlgrenska University Hospital, Göteborg, Sweden; 3 The Wallenberg Laboratory for Cardiovascular Research, Göteborg, Sweden; 4 Institute of Biomedicine, Sahlgrenska Academy, University of Gothenburg, Göteborg, Sweden; Midwestern University, UNITED STATES

## Abstract

**Background:**

The main inhibitor of the fibrinolytic system, Plasminogen Activator Inhibitor -1 (PAI-1), irreversibly binds tissue-type Plasminogen Activator (t-PA) and thereby inhibits the protective action of tPA against thrombus formation. Elevated levels of plasma PAI-1 are associated with an increased risk of cardiovascular events and are observed in subjects with type 2 diabetes (T2D) and obesity. Platelets contain the majority of PAI-1 present in blood and exhibit the ability to synthesis active PAI-1. Diabetic platelets are known to be hyper-reactive and larger in size; however, whether these features affect their contribution to the elevated levels of plasma PAI-1 in T2D is not established.

**Objectives:**

To characterize the PAI-1 antigen content and the mRNA expression in platelets from T2D subjects compared to obese and lean control subjects, in order to elucidate the role of platelet PAI-1 in T2D.

**Methods:**

Nine subjects with T2D and obesity were recruited from Primary Care Centers together with 15 healthy control subjects (8 lean subjects and 7 with obesity). PAI-1 antigen levels in plasma, serum and platelets were determined by ELISA, and PAI-1 mRNA expression was analyzed by qPCR.

**Results:**

There was no significant difference in PAI-1 mRNA expression or PAI-1 antigen in platelets in T2D subject in comparison to obese and lean control subjects. An elevated level of plasma PAI-1 was seen in both T2D and obese subjects. PAI-1 gene expression was significantly higher in both obese groups compared to lean.

**Conclusion:**

Similar levels of protein and mRNA expression of PAI-1 in platelets from T2D, obese and lean subjects indicate a limited role of platelets for the elevated plasma PAI-1 levels. However, an increased synthesis rate of mRNA transcripts in platelets from T2D and an increased release of PAI-1 could also result in similar mRNA and protein levels. Hence, synthesis and release rates of PAI-1 from platelets in T2D and obesity need to be investigated to further elucidate the role of platelets in obesity and T2D.

## Introduction

Type 2 diabetes (T2D) and the metabolic syndrome (MetS), which is a combination of metabolic features including obesity, are two of the major public health challenges of the 21^st^ century [1–5]. These conditions are associated with an increased risk of cardiovascular disease, and thrombotic events are the most common cause of death [6, 7]. There is emerging evidence suggesting an important role of an impaired endogenous fibrinolytic system; plasminogen activator inhibitor -1 (PAI-1) has become recognized as a central molecule linking the MetS to thrombotic vascular events [8, 9].

PAI-1 is the main inhibitor of the fibrinolytic system. In case of a thrombotic event, the intravascular fibrinolytic system is activated by an acute release of tissue-type plasminogen activator (t-PA) from endothelial cells, which by converting plasminogen to plasmin degrades fibrin into soluble products ensuring degradation of the thrombus. PAI-1 binds irreversibly and inhibits t-PA, and increased plasma levels of PAI-1 lead to a state of hypofibrinolysis in which the removal of thrombi is impaired [10]. In line with this, increased levels of plasma PAI-1 may cause both arterial and venous thrombosis [11, 12], whereas absence or deficiencies of PAI-1 result in excessive bleeding.

Absence or deficiencies of PAI-1 results in excessive bleeding [13, 14], on the contrary, PAI-1 is a strong predictor of myocardial infarction, although the predictive ability disappears after adjustment for markers of the MetS [15–18]. The association between PAI-1 and MetS including obesity is well established [19]. The elevated plasma PAI-1 levels in obese non-diabetic control subjects may be normalized by removal of adipose tissue either by dieting, surgical resection or bariatric surgery [20–24]. It is well known that T2D have increased plasma PAI-1 levels and there is a strong association between PAI-1 and T2D, independently of common diabetes risk factors [25–27].

The largest pool of PAI-1 in blood is stored in alpha granules in platelets and they contain approximately 90% of all circulating PAI-1 [28]. Platelets contain megakaryocyte-derived mRNA and the machinery to translate proteins; PAI-1 is synthesized *de novo* by platelets and the rate is approximately 35 fold greater than that required to maintain steady state plasma levels [29–31]. PAI-1 is synthesized in several different tissues *in vitro*, but to what extent these tissues contribute to plasma PAI-1 levels has been widely discussed [32]. By studying glycosylation patterns on plasma PAI-1, we have shown that platelets may be a major source of plasma PAI-1 in healthy humans, since there were no glycans either on PAI-1 in plasma or in platelets [33]. However, plasma PAI-1 in obese non-diabetic subjects possessed similar glycosylation patterns as PAI-1 from adipose tissue, suggesting a significant contribution of adipose tissue-derived PAI-1 in obesity.

Mean platelet volume (MPV) is an independent predictor of vascular events, and it is increased in patients with T2D and MetS [34, 35]. Furthermore, there is a positive correlation between HbA1c and MPV. Additionally, the larger platelets in patients with T2D are hyperreactive with increased aggregation, adhesion, and activation [36, 37]. There are some? studies indicating that the PAI-1 antigen content in platelets from T2D patients is reduced compared with healthy participants [38]. However, if the size and activity of platelets in T2D influence their ability to synthesize PAI-1 is still unknown.

Likewise, whether hyperreactive and hypertrophic platelets contribute to the increased levels of plasma PAI-1 in T2D is not established. As a first step to elucidate the role of platelet PAI-1 in T2D and obese non-diabetic control subjects the objective of this study was to characterize the PAI-1 antigen content as well as the mRNA expression levels in platelets from T2D patients compared with platelets from obese and lean healthy subjects.

## Material and methods

### Study participants

Eight participants with T2D and obesity (BMI ˃30 kg/m^2^) were recruited from Primary Care Centers together with 15 healthy control subjects, of which 7 were obese (BMI ˃30 kg/m^2^) and 8 were lean (BMI 18-25kg/m^2^). The study was performed in connection with the MD-lipolysis study, and some volunteers participated in both studies [39]. Inclusion criteria were; age 50–70 years for men and 55–70 years for women; postmenopausal state for women (defined as serum follicle stimulating hormone (s-FSH) > 26 mU/L); and duration of T2D less than 5 years. Both lean and obese non-diabetic control subjects were challenged with an oral glucose tolerance test (OGTT, 75 g) to ascertain normal glucose tolerance. Exclusion criteria were significant cardiovascular disease (ischemic heart disease or heart failure equal to NYHA II) not including hypertension with mono therapy; smoking; significant complications from T2D; treatment with glucagon-like protein-1 (GLP-1) agonists, insulin, beta blockers, glitazones, or dipeptidyl peptidase-4 (DPP-4) inhibitors; hematological diseases or bleeding disorders; and other concomitant disease or complications of significance as determined by medical history, physical examinations or screening laboratory evaluations. Pharmacological treatment was withdrawn 10 days prior to the study, and the participants were attending an extra visit 3–4 days prior to the measurement of fP-glucose and blood pressure. All participants were asked to avoid exercise and alcoholic beverages 48h prior to the visit, eat a light meal the evning before the visit, and stay fasting after midnight. According to the Helsinki declaration, all participants signed a written informed consent prior to participation. The protocols were approved by the Ethical Committee of the University of Gothenburg (Dnr 844–12).

### Chemical analysis

All basic chemical analyses of venous blood were conducted at the Laboratory for Clinical Chemistry at Sahlgrenska University Hospital (Gothenburg, Sweden). Mean platelet volume (MPV) was analyzed using Celldyn Sapphire (Abbott, US).

### Platelet, plasma and serum isolation

Human blood was drawn in acid-citrate-dextrose (ACD 1.5ml/8.5ml) tubes containing 100 nM prostaglandin E1 (PGE_1_) (Sigma, St Louis, USA) in a total of 100ml from each subject between 9 and 10 am. Platelet-rich plasma (PRP) was prepared, with minimal leucocyte contamination, as previously described [29]. In brief, after centrifugation of the blood samples at 150xg for 20 min at room temperature (RT), platelet rich plasma (PRP) was carefully removed and re-centrifuged at 150xg for 10 min. The platelets were pelleted at 800xg for 15 min and subsequently resolved in Trizol LS (Ambion by Life Technologies, Paisley, Scotland, UK) and stored in -80°C until mRNA isolation. Platelet poor plasma was re-centrifuged at 2000xg for 20 min at 4°C to remove residual platelets and stored in -80°C until analysis. To analyze PAI-1 content in platelets, 500μl PRP was centrifuged at 2000xg for 20 min and subsequently re-suspended in lysis buffer (0.1% Triton^®^ X-100 (Roche Diagnostics GmbH, Penzberg, Germany), 1% BSA (Sigma-Aldrich) in PBS (Lonza) at pH 7.4). After lysis for 30 minutes on ice the platelet lysate was centrifuged at 10 000xg for 10 minutes at 4°C to remove cell debris. The supernatant of platelet lysate was diluted 1:10 in 1% BSA in PBS prior to protein analysis. Human blood was drawn in one SST^™^-tube, centrifuged at 2000xg for 20 min and prepared serum was stored in -80°C until analysis. Additionally, 3 ml blood was collected in Hirudin blood tubes (Roche Diagnostics) and platelet aggregation ability was measured by Multiplate^®^ analyzer (Roche Diagnostics). TRAP-test (Thrombin receptor activating peptide-6), ASPI-test (arachidonic acid) and ADP-test (adenosinediphosphate) were used in final concentrations of 32μM, 0.5mM and 6.5mM respectively. The Multiplate^®^ registers adhesion and aggregation by multiple electrode aggregometry, which is expressed via the area under the curve in arbitrary units.

### Subcutaneous adipose tissue isolation

Subcutaneous adipose tissue was collected from the periumbilical region by needle aspiration, under local anesthetic blockade (Carbocain 10 mg/ml without adrenaline, Aspen Nordic, Ballerup, Denmark). Adipose tissue was immediately washed in warm PBS with forceps, snap-frozen in liquid nitrogen and stored in -80°C until RNA extraction.

### Protein analysis

Plasma, serum, and platelet PAI-1 antigen was determined by an Enzyme-Linked Immuno Sorbent Assay (ELISA) (Technoclone GmbH, Vienna, Austria) according to manufacturer’s instructions. The results of platelet PAI-1 antigen presented was calculated based on manually counted platelets in PRP using microscope.

### Gene expression analysis

RNA in platelets was extracted by using Trizol LS according to manufactures instructions. In adipose tissue, RNeasy Lipid Tissue Mini Kit (Quiagen, Santa Clara, Calif., USA) was used according to the manufacturer’s instructions. Total RNA concentrations were determined on Qubit 2.0 Fluorometer using Qubit RNA HS Assay Kit according to manufacturer’s instructions (Life Technologies, Eugene, Oregon, USA). Reverse transcription was carried out in 0.25 μg of RNA in a total volume of 20 μl reaction mixture, High-capacity RNA-to-cDNA master mix (Applied Biosystems, Foster City, CA, USA). Samples were incubated at 37°C for 60 minutes, at 95°C for 5 minutes and finally at 4°C. Relative quantification was performed on 7500 Fast Real-Time PCR System (Applied Biosystems). For amplification of the candidate genes, 0.24 μl cDNA was added to the PCR mixture consisting of TaqMan Universal PCR Master Mix Fast, in a final volume of 15 μl (Applied Biosystems). By using the Primer Express version 1.0 software (Applied Biosystems) primers and probes of PAI-1 was designed and were used in a concentration of 0.4 μM and 0.2 μM. Each primer pair was selected so that the amplicon spanned an exon junction to preclude amplification of genomic DNA. By using a dilution curve, the efficiencies of the home-designed primer and probes were validated (data not shown).

YWHAE and B2M were evaluated as stable reference genes in platelets by using the software RefFinder to correct for biological and technical errors, as previously described [40]. LPR10 has previously been evaluated as a stable reference gene in human adipose tissue [41]. Pre-designed primers and TaqMan probes for references genes YWHAE, B2M (Gene Expression Assays, Applied Biosystems) and LRP10 (Life Technologies) for the reference genes were used (Table 1).

**Table 1:** Primers and probes for qPCR.

Gene symbol	Gene title	Sequence	Position	Gene Expression Assay
**PAI-1**	Plasminogen Activator Inhibitor -1			
forward primer		5’-ggc tga ctt cac gag tct ttc a-3’	11616–11637	
reverse primer		5’-ttc act ttc tgc agc gcc t-3’	11758–11780	
probe		5’-(FAM)acc aag agc ctc tcc acg tcg cg (TAMRA)-3’	11782–11800	
**YWHAE**	Tyrosine 3-monooxygenase/tryptophan 5-monooxygenase activation protein			Hs00356749_g1
**B2M**	Beta-2-microglobulin			Hs00187842_m1
**LRP10**	LDL receptor related protein 10			Hs00204094_m1

### Data analysis

Raw Ct-values were normalized to the two reference genes YWHAE, B2M and LRP10 respectively (dCt) and transformed by using the equation 2^-ddCt, then normalized to the mean of the lean control group (ddCt). In order to stabilize variances and to get symmetrical distributions for the residuals all statistical analyses of PAI-1 were performed on log transformed data. Results were analyzed using SPSS statistical software (SPSS Inc, Chicago, IL). Variance analysis ANOVA with post-hoc Bonferroni correction was used to analyze differences in means between groups. Pearson correlation coefficients were calculated to determine associations. Results were considered significant if *p* ˂ 0.05.

## Results

### Characteristics of the study participants

The characteristics of the three subject groups are shown in Table 2. All participants were well-defined regarding obesity; BMI and waist circumference differed significantly between lean control subjects, and T2D patients or obese non-diabetic control subjects (*P*˂0.001 respectively), and waist-hip-ratio (WHR) differed significantly between T2D and lean controls (*P =* 0.008). Moreover, T2D patients were distinctly differentiated from the two control groups concerning factors associated with diabetes. HbA1c and fP-glucose were higher in T2D patients compared with obese and lean control subjects (*P*˂0.001 respectively) and the oral glucose tolerance test (OGTT) was normal in obese and lean control subjects (data not shown). Furthermore, there were significant differences between the groups regarding characteristics associated with the MetS; higher blood pressure, heart rate and ALAT were observed in T2D patients compared with lean control subjects and significantly increased levels of triglycerides and decreased levels of HDL were observed in both T2D patients and obese non-diabetic controls compared with lean control subjects.

**Table 2:** Characteristics of the study participants.

	T2D	Obese	Lean	*p*-value
**Sex** (M/F)	4/4	2/5	3/5	NS
**Diabetes duration**	3.2±1.2			
**Age** (years)	59.5±5.6	60.4±5.6	60.1±5.8	NS
**BMI** (kg/m^2^)	34.2±2.7*	32.4±1.9#	23.2±2.1	˂0.001
**Waist** (cm)	112.8±7.0*	110.4±5.5#	83.7±10.7	˂0.001
**WHR**	0.97±0.08*	0.94±0.07	0.84±0.09	0.008
**SBP** (mmHg)	154.5±14.2*	135.3±18.1	117.1±12.4	˂0.001
**DBP** (mmHg)	94.1±6.8*	86.4±10.6#	74.1±9.30	0.001
**fP-Glucose** (mmol/l)	9.3±2.50*,Ψ	5.6±0.55	5.0±0.77	˂0.001
**B-HbA1c** (mmol/mol)	54.3±8.6*,Ψ	35.6±3.4	32.6±1.8	˂0.001
**S-Cholesterol** (mmol/l)	5.2±1.0	5.2±1.2	5.5±1.2	NS
**S-Triglycerides** (mmol/l)	1.6±0.51*	0.38±0.14#	0.18±0.06	0.001
**S-HDL-c** (mmol/l)	1.3±0.28*	1.2±0.27#	1.9±0.42	0.001
**S-LDL-c** (mmol/l)	3.6±1.00	3.7±0.99	3.6±0.97	NS
**S-Creatinine** (μmol/)	70.5±7.1Ψ	88.3±7.9	78.8±13.1	0.008
**S-ALAT** (μkat/L)	0.77±0.33*,Ψ	0.47±0.12	0.33±0.06	0.001

Data presented as mean±SD. One-way ANOVA was used for statistical analyses, with Post Hoc test Bonferroni;

*significance difference between control and T2D,

^#^significance difference between control and obese, and

^Ψ^significance difference between obese and T2D.

Abb. BMI: Body mass index; WHR: Waist-hip ratio; SBP: systolic blood pressure; DBP; diastolic blood pressure; B-HbA1c: Glycated hemoglobin; hs-CRP: high sensitivity C-Reactive Protein; S-HDL-c: High-density lipoprotein; S-LDL-c: Low-density lipoprotein; ALAT: Alanine transaminase; Hb: haemoglobin; LPK: leucocytes.

### Characteristics of platelets of diabetic patients

Table 3 summarizes the characteristics of platelets from T2D patients compared with those of obese and lean control subjects. Platelet counts were similar between the groups (*P =* 0.621). The mean platelet volume (MPV) was decreased by 19% in T2D compared with obese non-diabetic control subjects (*p =* 0.019), whereas no other significant difference between the groups was observed. MPV was not correlated to BMI, plasma glucose or HbA1c. Platelet aggregation ability, measured by multiplate analyzer, was within reference values in all groups. No differences between the groups were detected; with the exception for ASPI-test where obese non-diabetic controls had an increased aggregation ability in response to arachidonic acid compared with lean control subjects (*p =* 0.005).

**Table 3:** Platelets in T2D patients compared with obese and lean control subjects.

	T2D	Obese	Lean	*p*-value
**B-PLT** (x10^9^/L)	256.8±60.6	238.4±51.2	232.4±39.6	NS
**B-MPV** (fL)	7.3±0.84Ψ	9.0±0.58	8.5±1.20	0.020
**P-TRAP**	118.3±13.5	134.8±22.9	105.4±15.7	NS
**P-ADP**	78.3±26.6	86.8±9.7	67.0±23.9	NS
**P-ASPI**	91.7±9.5	107.0±5.7#	84.0±12.2	0.005

Data presented as mean±SD. One-way ANOVA was used for statistical analyses, with Post Hoc test Bonferroni;

*significance between control and T2D,

^#^significance between control and obese, and

^Ψ^significance between obese and T2D.

For TPK and MPV; T2D n = 8, obese n = 7, lean n = 8. For multiplate results; T2D n = 3, obese n = 6, lean n = 5. Abb. PLT: platelet count; MPV: mean platelet volume; TRAP: thrombin receptor activating peptide-6; ADP: arachidonic acid; ADP-test: adenosinediphosphate.

### PAI-1 antigen levels in plasma, serum, and platelets

**Fig 1:**
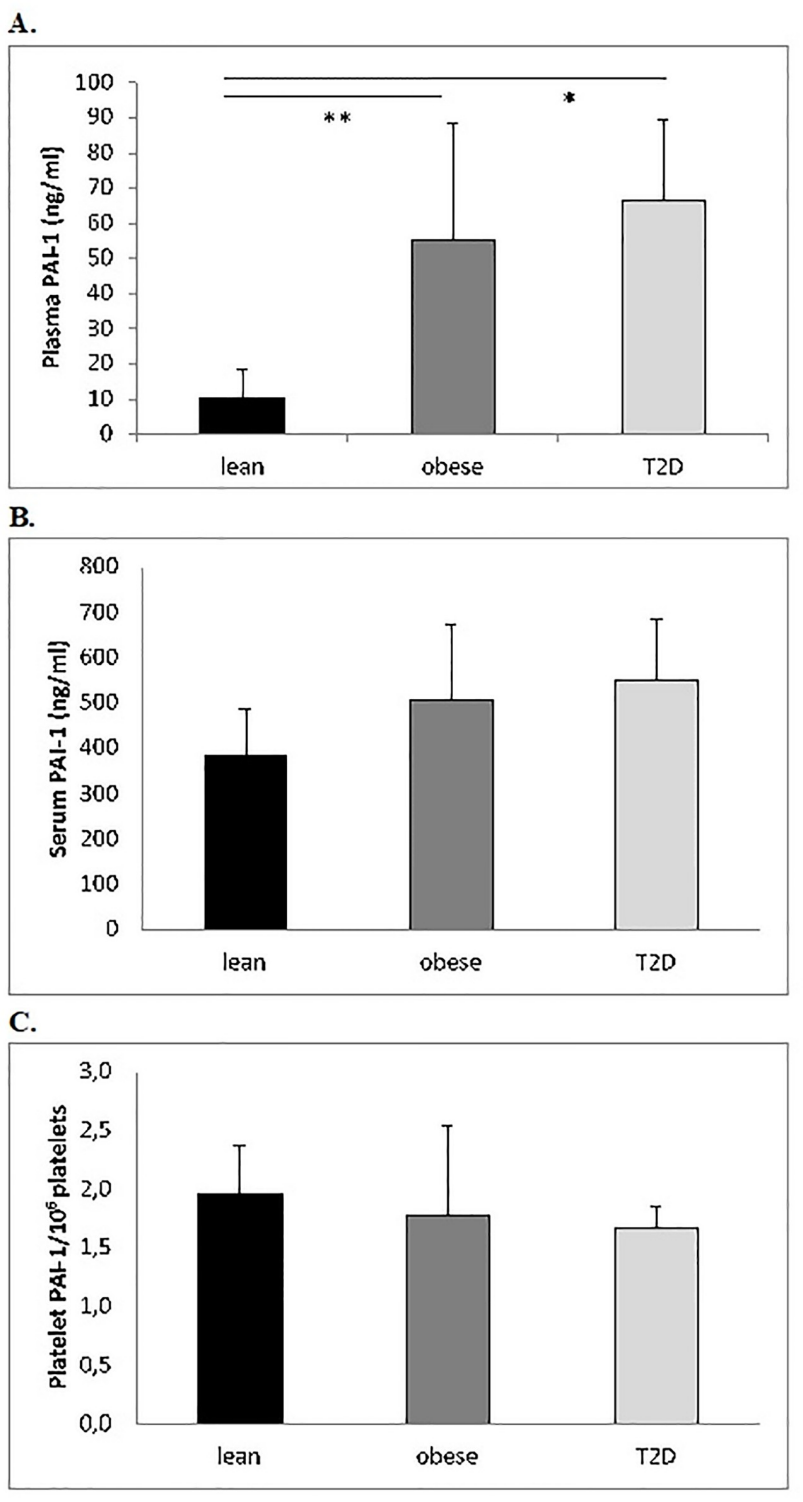
Pool of PAI-1 in A. plasma, B. serum and C. platelets in T2D patients, lean and control participants. There was a significant increase of plasma PAI-1 in participants with T2D and obese non-diabetic control subjects compared with lean control subjects (T2D n = 6, obese n = 7 and lean control subjects n = 8). * *P*˂0.001, ** *P*˂0.001. B. No significant difference in serum PAI-1 between the three groups (T2D n = 6, obese n = 7 and lean control subjects n = 8). C. There was no significant difference in platelet PAI-1 between the three groups (T2D n = 4, obese n = 5 and lean control subjects n = 8). Abb. PAI-1 = plasminogen activator inhibitor -1; T2D = type 2 diabetes.

As expected, there was a statistically significant difference in plasma PAI-1 levels between the three groups (*p*˂0.001), as illustrated in Fig 1A. There was an approximately 6-fold increase in T2D patients and obese non-diabetic control subjects to 66.5±22.8 and 55.1±33.1 ng/ml respectively compared with lean control subjects 10.4±8.3 ng/ml (*p*˂0.001 and *p*˂0.001), However, there was no significant difference between T2D patients and obese non-diabetic control subjects. A non-significant trend towards an increase in serum PAI-1 levels was observed comparing T2D (549.9±134.8 ng/ml), obese non-diabetic control subjects (508.2±164.9 ng/ml) and lean control subjects 383.75±104.54 ng/ml (*P =* 0.053) (Fig 1B). Additionally, there was no difference in the platelet pool of PAI-1 between the three groups; T2D 1.67±0.19 ng/10^6^ platelets, obese 1.77±0.76 ng/10^6^ platelets and lean control subjects 1.97±0.41 ng/10^6^ platelets (P = 0.606) (Fig 1C).

**Fig 2:**
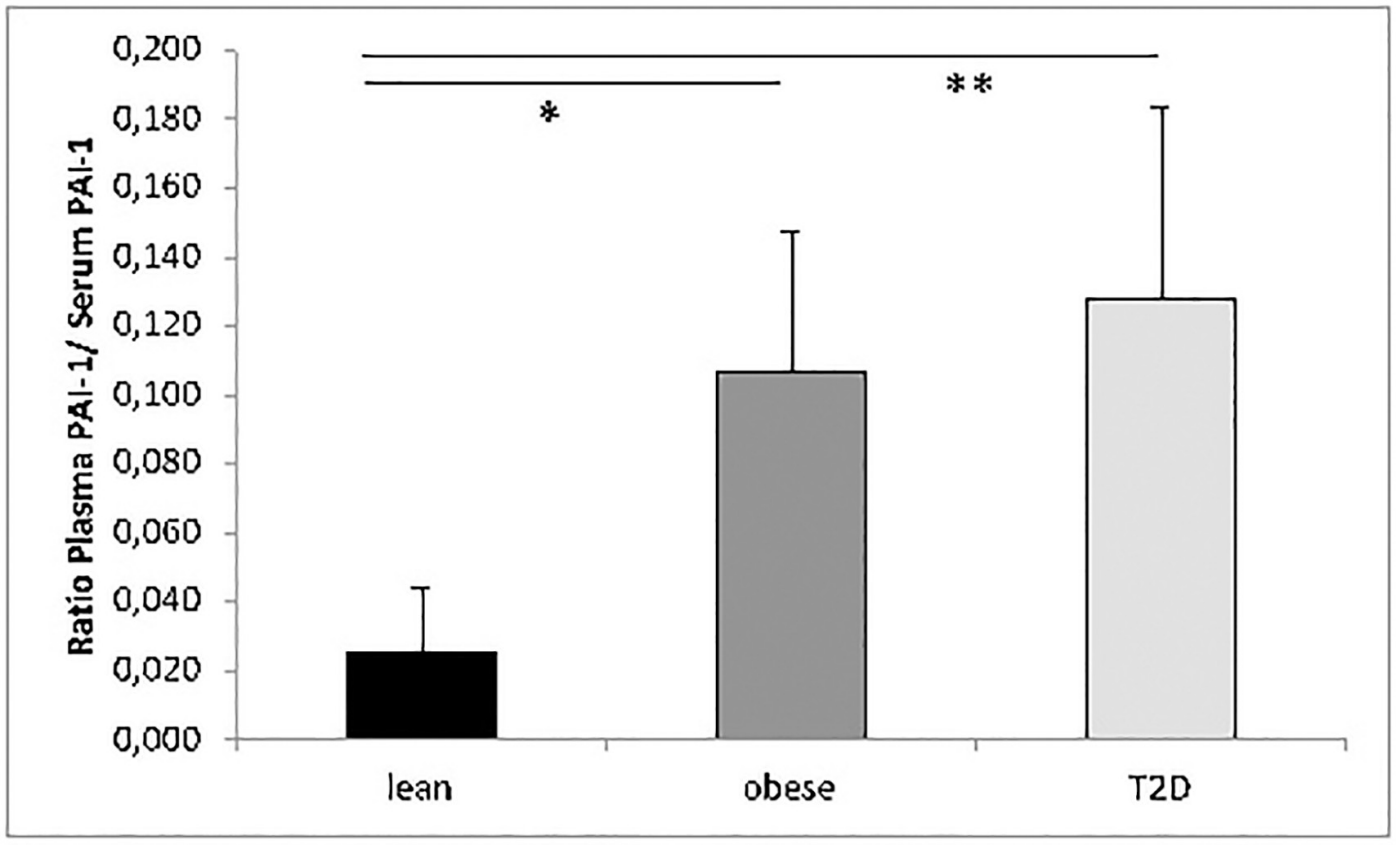
Ratio of plasma PAI-1/serum PAI-1 in T2D. There was a significant difference in ratio between lean control subjects and T2D or obese non-diabetic control subjects (T2D n = 6, obese n = 7 and lean control subjects n = 8). * *P*˂0.001, ** *P*˂0.003. Abb. PAI-1 = plasminogen activator inhibitor -1; T2D = type 2 diabetes.

There was a significant difference in the ratio between plasma and serum PAI-1 comparing T2D patients 0.13±0.056 or obese non-diabetic control subjects 0.12±0.041 to lean control subjects 0.025±0.019 (P˂0.001 and P = 0.003 respectively) shown in Fig 2.

Plasma PAI-1 correlated significantly to BMI and waist circumference (r = 0.83, *P*˂0.001 and r = 0.78, *P*˂0.001 respectively) and to factors involved in the MetS; P-glucose, HbA1c and triglycerides (r = 0.54, *P =* 0.011; r = 0.54, *P =* 0.011; r = 0.67, *P =* 0.001). By contrast, there was no significant correlation to platelet count or MPV. Plasma PAI-1 and serum PAI-1 correlated significantly (r = 0.67, *P =* 0.001), but not to platelet PAI-1.

### Gene expression of PAI-1 in platelets

**Fig 3:**
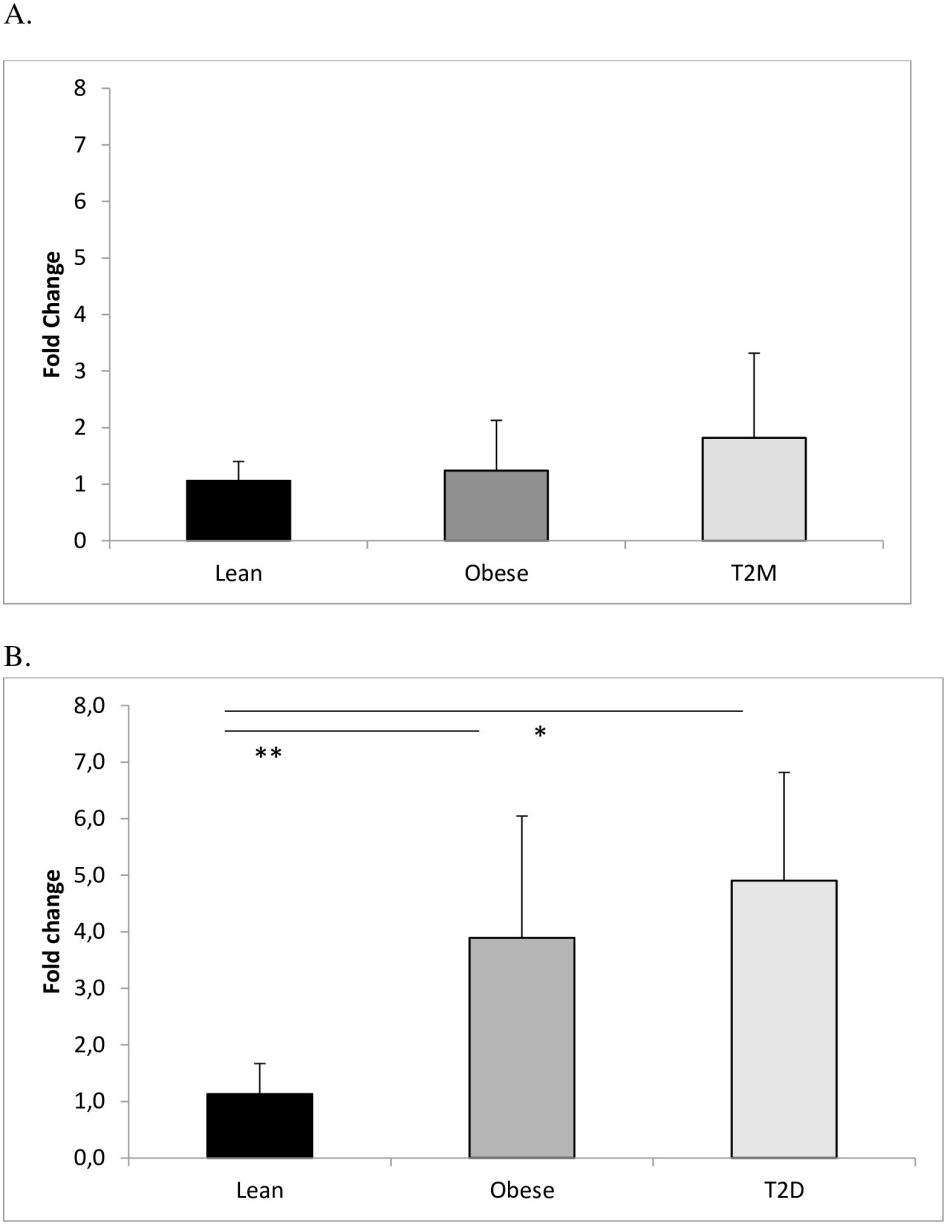
Gene expression levels of PAI-1 in A. platelets compared with reference genes and B. in subcutaneous adipose tissue compared with reference genes. There were no significant differences in gene expression levels of PAI-1 in platelets between participants with T2D, obese and lean control subjects (T2D n = 6, obese n = 7 and lean control subjects n = 7)(A.). There was a significant difference in gene expression levels of PAI-1 in subcutaneous adipose tissue between lean control subjects and T2D or obese non-diabetic control subjects (T2D n = 7, obese n = 6 and lean control subjects n = 6) * *P =* 0.003, ** *P =* 0.038 (B.). Abb. PAI-1: Plasminogen Activator Inhibitor -1; T2D: type 2 diabetes.

The mRNA levels of PAI-1 in platelets are shown in Fig 3. There was no difference in gene expression levels of PAI-1 between T2D, obese and lean control subjects normalized to YWHAE or B2M (*P =* 0.558 and *P =* 0.332 respectively).

### Gene expression of PAI-1 in subcutaneous adipose tissue

**Fig 4:**
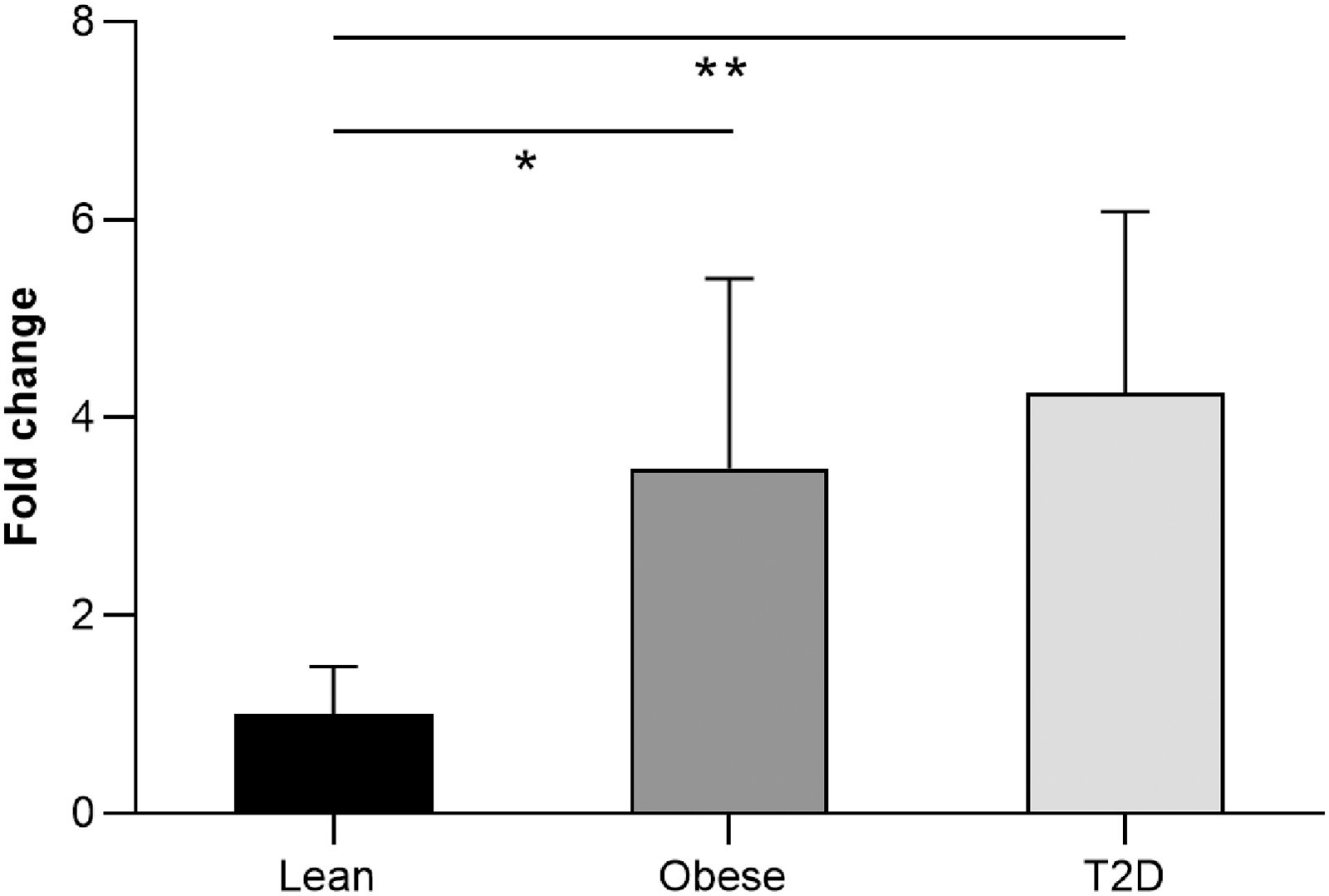
Gene expression of PAI-1 in subcutaneous adipose tissue of lean, obese and T2D normalized against LRP10. ANOVA with Bonferroni’s multiple comparisons test. N = 6 for all groups. Abb. PAI-1: Plasminogen Activator Inhibitor -1; T2D: type 2 diabetes.

There was a statistically significant difference in gene expression levels of PAI-1 in subcutaneous adipose tissue between the three groups (P = 0.007) (Fig 4). There was an approximately 4-fold increase in T2D patients and 3.5-fold increase in obese non-diabetic control subjects compared with lean control subjects (P = 0.008 and P = 0.044 respectively); but there was no significant difference between T2D and obese non-diabetic control subjects.

## Discussion

In the present study we characterized the pool of PAI-1 in platelets in T2D patients and diabetes-free control subjects, to evaluate their contribution to circulating PAI-concentrations in plasma. As expected, we found increased plasma PAI-1 levels both in T2D patients and healthy obese subjects. Furthermore, there were highly significant correlations between BMI or triglycerides and plasma PAI-1, indicating that participants in our groups were representative [42–44]. In addition, we confirmed that PAI-1 mRNA expression is increased in abdominal subcutaneous adipose tissue both in T2D and obese non-diabetic subjects compared with lean control subjects. However, the pool of PAI-1 as well as PAI-1 mRNA expression in platelets were similar among the groups and this suggests that other sources than platelets determine the increased plasma PAI-1 level in T2D patients.

Platelet hyperreactivity plays a critical role in the pathogenesis of atherothrombosis in patients with obesity and/or T2D and these conditions are also associated with an increased MPV [34, 36, 37, 45]. These studies indicate that the platelet mRNA expression and the synthesis of PAI-1 could be altered in obesity and T2D. However, in this study, no significant difference in PAI-1 mRNA expressions in platelets between the groups was observed. Moreover, platelet PAI-1 antigen levels were similar among the groups, in contrast to previous studies which have observed a reduction in platelet PAI-1 in T2D patients [38]. There was a significant increase in PAI-1 gene expression in abdominal subcutaneous adipose tissue in T2D and obesity, compared with lean healthy subjects. Similarly, PAI-1 mRNA expression has previously been reported to be upregulated in adipose tissues in obese mice and humans [46, 47]. Thus, subcutaneous adipose tissue may be more important than platelets for PAI-1 levels in plasma in obese T2D patients.

The significant difference in ratio plasma/serum PAI-1 between lean controls and T2D patients or obese non-diabetic subjects may indicate a different source of PAI-1 or increased platelet contribution to plasma PAI-1. A higher synthesis rate and turnover of PAI-1 in platelets from T2D patients compared with obese and lean healthy subjects could not be ruled out. Further studies need to confirm if platelets in T2D initially contain more PAI-1 mRNA transcripts that are translated into protein at an increased rate and followed by a direct release of PAI-1 into the blood stream.

Platelet count and analyses of platelet aggregation were not changed in T2D patients compared with healthy obese and lean subjects. However, obese non-diabetic controls exhibited significantly larger platelets than T2D patients, and platelet aggregation induced by arachidonic acid was increased in obese compared to lean non-diabetic control subjects. The reason for these findings is unclear. Otherwise, the platelet phenotype was relatively unchanged between the three groups. Several studies have previously shown that platelets from T2D patients exhibit an abnormal functional profile, although most of these studies revealed a high glycemic burden or significant diabetic complications among the participants included [36, 48, 49]. Conversely, Shlomai et al. have demonstrated normal platelet size and function in T2D patients showing good metabolic control and without prior ischemic events [50]. The T2D patients in this study were also well controlled and that may in part explain our neutral findings in PAI-1 expression in platelets. In addition, there is evidence that MPV values decrease in patients who achieve improved metabolic control [35].

A potential limitation of this study is the relatively low number of participants with a risk for type II errors. Furthermore, T2D patients of this cohort had relatively short diabetes duration, and it is possible that studies on diabetes patients with poor metabolic control or vascular complications could show different results. On the other hand, included patients had no late complications, were well characterized and all drugs were suspended for 10 days prior to the study procedures to avoid interference with concomitant medication. Thus, the mild and recently diagnosed diabetes phenotype studied in this article reduces the influence of confounding factors to the analysis.

In conclusion, we have for the first time quantified mRNA expression of PAI-1 in platelets from T2D patients. Characterization of the pool of PAI-1 in platelets from T2D patients revealed that there is no significant difference in platelet PAI-1 compared with obese and lean individuals. To further elucidate the role of platelets in obesity and T2D, synthesis and release rates of PAI-1 from platelets need to be investigated.

## Supporting information

S1 Data (XLSX)
